# Maternal and perinatal characteristics associated with congenital anomalies: a case-control study

**DOI:** 10.61622/rbgo/2025rbgo44

**Published:** 2025-07-15

**Authors:** Andreia Helena Scandolara, Raquel Maiéli Bagatini, Ana Luiza Goulart Starck, Ricardo Babinski Bregonde, Claudicéia Risso Pascotto, Fernando Rodrigo Treco, Alessandro Rodrigues Perondi, Lirane Elize Defante Ferreto

**Affiliations:** 1 Universidade Estadual do Oeste do Paraná Francisco Beltrão Paraná Brazil Universidade Estadual do Oeste do Paraná, Francisco Beltrão, Paraná, Brazil.; 2 Universidade Paranaense Francisco Beltrão Paraná Brazil Universidade Paranaense, Francisco Beltrão, Paraná, Brazil.

**Keywords:** Congenital abnormalities, Infant, newborn, Pregnancy, Primary prevention, Perinatal care, Maternal and child health

## Abstract

**Objective:**

This study aimed to identify the patterns and factors associated with congenital anomalies in newborns from a tertiary hospital maternity ward.

**Methods:**

A case-control study was conducted at the Hospital Regional do Sudoeste Walter Alberto Pecóits in Francisco Beltrão (PR), between December 2023 and September 2024. Among the 1,400 births that occurred in the hospital's delivery room between December 1, 2023, and the end of September 2024, 37 newborns (2.6%) with congenital anomalies were identified during the study period. A total of 37 case mothers and 120 controls were included (a ratio of approximately 1:3). The sociodemographic variables included maternal age, residence, marital status, race, education level, family income, and maternal occupation. The behavioral variables considered smoking, alcohol consumption, and the use of drugs or medications during pregnancy. The fetal variables included the number of pregnancies, gestational age, type of delivery, miscarriage, Apgar score, birth weight, sex, fetal status, congenital anomalies, and the number of prenatal visits, with a minimum of six (one in the first trimester, two in the second, and three in the third), following the Ministry of Health guidelines.

**Results:**

Among 1,400 live births, 37 cases of congenital anomalies were identified (2.6%, 95% CI: 1.80–3.48), while the control group included 120 women with newborns without congenital anomalies. Among cases, isolated anomalies were most common (62.2%), predominantly affecting the cardiovascular system (27.0%), followed by recognized syndromes (21.6%) and multiple malformations (16.2%). Data were collected through face-to-face interviews and medical record reviews. Bivariate analysis revealed significant associations between congenital anomalies and a family history of congenital anomalies (p = 0.02), low apgar scores at the 1st and 5th minutes (p < 0.01), and fetal status at birth (p < 0.01). Model 1, which integrates family history of congenital anomalies, apgar score at the 5th minute, and fetal status, showed the best predictive fit, consistent with previous findings. Bayesian logistic regression highlighted this model with the lowest AIC (295.98) and BIC (326.22) values, achieving 89% predictive accuracy.

**Conclusion:**

These results reinforce the importance of family history and neonatal vitality in the context of congenital anomalies, indicating the need for future studies to confirm these findings and improve prevention strategies.

## Introduction

Congenital anomalies (CAs) are the second leading cause of infant mortality and are considered a public health priority, with a higher prevalence in low- and middle-income countries.^(1–3)^ These conditions are still often perceived by the medical community as rare and, in many cases, inevitable events.^(3,4)^

Approximately 50 to 60% of CAs have an unknown etiology, representing a biologically heterogeneous group of embryofetal developmental disorders with distinct etiological factors often acting simultaneously.^(2,5)^ Among the recorded cases, 15 to 20% are associated with monogenic or chromosomal genetic conditions, 20% have a multifactorial etiology, and 7% are related to congenital infections and exposure to teratogens, including congenital infections and the use of medications, alcohol, and illicit drugs in Brazil.^(1,6)^

In Brazil, the prevalence of CAs ranges from 2% to 5% of live births, which is very close to that observed in other regions of the world. The main risk factors associated with CAs include a family history of congenital malformations, maternal alcohol consumption, gestational diabetes, and previous miscarriages. Additionally, as observed in other countries, there is an association between CAs and maternal exposure to pesticides.^(2,3,6)^ The occurrence of any CA has an immeasurable impact on families’ lives and infant mortality rates, accounting for 30% to 50% of perinatal fetal deaths and 20% of neonatal deaths in some countries. In Brazil, they are the second leading cause of infant mortality in all regions. The impact of CAs on infant mortality depends on factors such as the prevalence of anomalies, access to medical and surgical treatment, and primary prevention measures.^(5,7)^

It is important to highlight that beyond the financial burden caused by prolonged hospitalizations and multiple corrective surgeries, there is an increased risk of clinical complications, directly impacting the quality of life of affected children and their families.^(3,8,9)^

In this context, the present study aimed to determine the pattern and factors associated with congenital anomalies in newborns from a tertiary hospital maternity ward.

## Methods

The study was conducted at the Hospital Regional do Sudoeste Walter Alberto Pecóits, located in the municipality of Francisco Beltrão, Paraná, Brazil. This tertiary hospital is the only one in the Southwest region of Paraná responsible for providing care for high-risk pregnancies, offering services to 27 municipalities and serving a population of approximately 333.967 inhabitants, based on the sum of the estimated populations of these municipalities, according to data from the Brazilian Institute of Geography and Statistics (IBGE).^(10)^

**Figure 1:**
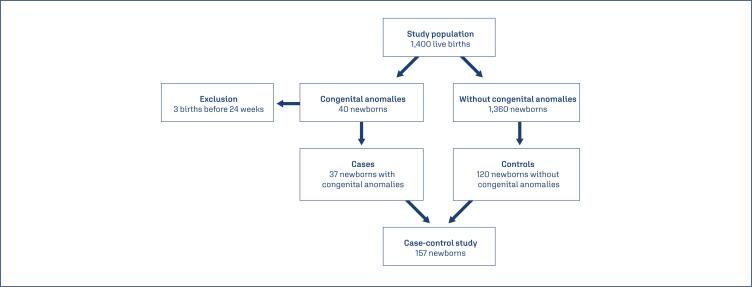
Flowchart of the case-control study population

Among the 1,400 births that occurred in the hospital's delivery room between December 1, 2023, and the end of September 2024, 37 newborns (2.6%) with congenital anomalies were identified during the study period. A total of 37 case mothers and 120 controls were included (a ratio of approximately 1:3). All women who gave birth to babies with congenital anomalies during the study period were included. The control group consisted of two to three women who gave birth to babies without congenital anomalies, recruited on the same day as a case mother. The inclusion criterion was the birth of babies without congenital anomalies identified on the same day as those in the case group. Women of any age and parity were included, with spontaneous vaginal deliveries, induced deliveries, or cesarean sections. A total of three women who gave birth before 24 weeks of gestation were excluded from the study (n = 3), as shown in figure 1.

The information about the women was collected through a questionnaire developed for the study,^(2,4,6,11)^ completed via face-to-face interviews after obtaining written informed consent.

The dependent variable in this study was the birth of babies with congenital anomalies at the Hospital Regional do Sudoeste Walter Alberto Pecóits, from December 1^st^, 2023, to the end of September 2024. The independent variables were classified into sociodemographic, behavioral, maternal, fetal, and educational categories.

The sociodemographic variables included maternal age, residence, marital status, race, education level, family income, and maternal occupation. The behavioral variables considered smoking, alcohol consumption, and the use of drugs or medications during pregnancy. The maternal variables addressed pre-existing or gestational diseases, gestational weight, consanguinity, and family history of congenital anomalies.

The fetal variables included the number of pregnancies, gestational age, type of delivery, miscarriage, Apgar score, birth weight, sex, fetal status, congenital anomalies, and the number of prenatal visits, with a minimum of six (one in the first trimester, two in the second, and three in the third), following the Ministry of Health guidelines. Finally, the maternal education level was classified as illiterate, elementary education, high school, undergraduate, postgraduate, master's, and doctoral degrees.

All mothers of babies with congenital anomalies were included, diagnosed by the obstetrician during prenatal care or during delivery by the attending obstetrician or pediatrician during resuscitation. The control group consisted of women who gave birth to babies without congenital anomalies. The diagnosis of congenital anomalies was performed through routine ultrasound during regular pregnancy monitoring or identified at the time of delivery by the obstetrician or pediatrician using complementary examinations, following the hospital unit's protocol.

The researcher collected data from both groups, examined the women, and monitored the newborns in the neonatal intensive care unit or maternity ward, following the health unit's protocol. After obtaining formal consent, face-to-face interviews were conducted with the mothers. The mode of delivery was recorded for all women in both groups, and birth weight was obtained in the delivery room or from the baby's health record.

The descriptive analysis of participants was carried out using absolute numbers and frequencies for each category. To assess the relationship between the independent and dependent variables (congenital anomalies during pregnancy), the chi-square test (X^2^) was used. Subsequently, Bayesian logistic regression,^(12)^ was performed, testing models composed of variables that were significant (p ≤ 0.05) in the chi-square test (X^2^). Next, binary logistic regression (classification) was conducted for the selected models, considering 30% of test variables.^(13)^ Afterward, the possibility of variable moderation was assessed using the conditional logistic model.^(14)^ The significance level (p) used was ≤ 0.05.^(15,16)^ Statistical analyses were performed using Jeffreys's Amazing Statistics Program (JASP).^(17)^

The study was approved by the Human Research Ethics Committee of the State University of Western Paraná (UNIOESTE), under opinion number 6.505.092 and registered with *Certificado de Apresentação de Apreciação Ética* (CAAE) 74146623.5.0000.0107.

## Results

Among the 1,400 live births, 37 cases of congenital anomalies were identified at the time of delivery, representing a prevalence of 2.6% (95% CI: 1.80 - 3.48). Recognized syndromes accounted for 21.6% of cases, associations of two or more anomalies for 16.2%, and isolated anomalies for 62.2%, of which 27.0% predominantly involved the cardiovascular system, as shown in table 1.

**Table 1:** Types of congenital anomalies recorded in medical records at birth

Congenital anomalies			n(%)
Syndromes recognized			
		Boy Stalk	1(2,7)
		Edwards	5(13,5)
		Dandy-Walker	1(2,7)
		Tetralogy of Fallot	1(2,7)
Association of two or more anomalies			
		Multiple malformations	4(10,8)
		Cleft lip + Cleft palate	1(2,7)
		Congenital cluboot + Genetic syndrome	1(2,7)
Isolated anomalies			
	Cardiovascular system		
		Ventricular septal defect	5(13,5)
		Hydrops fetalis	1(2,7)
		Congenital heart disease	4(10,8)
	Cranium and central nervous system		
		Myelomeningocele	1(2,7)
	Facial malformations		
		Cleft lip	1(2,7)
		Anophthalmia	1(2,7)
	Gastrointestinal system		
		Omphalocele	1(2,7)
	Genitourinary system		
		Pyelectasis	2(5,4)
		Fetal hydronephrosis 4(6,8)	4(10,8)
	Musculoskeletal system		
		Congenital Clubfoot	1(2,7)
	Respiratory system		
		Respiratory system	1(2,7)
		Laryngomalacia	1(2,7)
Total			37 (100)

Abbreviations:%, frequency; n, sample.

The sample consisted of 157 women with a mean age of 29.40 years (Standard Deviation [SD] = 6.29). The mean age of the biological fathers was 32.76 years (SD = 8.46). The average family income was R$ 4,083.94 (SD = 3,233.23). The majority of women were in a stable union (50.96%, n = 80), self-identified as mixed race (46.68%, n = 78), and had incomplete or complete high school education (54.14%, n = 85). According to the bivariate analysis, receiving the COVID-19 vaccine during pregnancy, having siblings or grandparents with anomalies, number of pregnancies, Apgar score at the 1st minute, Apgar score at the 5th minute, and fetal status were significantly associated with congenital anomalies table 2.

**Table 2:** Relationship between congenital anomalies during pregnancy and the variables assessed

Assessed questions	Congenital anomalies during pregnancy					
	Case (n = 37)	Controls (n=120)				
	Average (DP)	Average (DP)	X^2^	GL	p-value	OR (IC95%)
Exposure to pesticides	2,00(0,00)	1,98(0,13)	0,56	1	0,45	6.181×10^-7^ (0,00-∞)
Tobacco use during pregnancy	1,97(0,17)	1,94(0,30)	1,15	2	0,56	0,55(0,10-1,08)
obacco use before pregnancy (former smoker)	1,97(0,30)	1,92(0,35)	0,59	2	0,75	0,65(0,20-2,14)
Alcohol use and abuse during pregnancy	1,97(0,17)	1,92(0,27)	1,10	1	0,29	0,34(0,04-2,77)
Illicit drug use and abuse during pregnancy	2,00(0,00)	1,97(0,18)	1,14	1	0,29	2,24×10^-7^(0,00-∞)
Medication use during pregnancy	1,00(0,00)	1,04(0,20)	1,43	1	0,23	4,51×10^+6^(0,00-∞)
Number of medications during pregnancy	2.59(0,12)	2,97(0,15)	7,85	8	0,45	1,23(0,91-1,66)
Folic acid supplementation	1,12(0,33)	1,09(0,29)	0,25	1	0,62	0,74(0,22-2,45)
Received COVID-19 vaccine during pregnancy	2,00(0,00)	1,64(0,67)	6,36	1	0,01	0,69(-0,81-0,59)
Received COVID-19 vaccine before pregnancy	1,00(0,00)	1,06(0,23)	2,04	1	0,15	4.627×10^+6^(0,00-∞)
Number of COVID-19 vaccine doses	2,65(0,85)	2,70(0,93)	1,10	4	0,89	1,07(0,69-1.66)
Presence of chronic diseases	1,697(0,47)	1,71(0,46)	0,01	1	0,91	1,05(0,46-2,43)
Presence of diseases during the gestational period	1,38(0,49)	1,37(0,48)	0,03	1	0,86	0,93(0,43-2,04)
Gestational weight	9.445,73(26,352,29)	2,641.44(14,083,48)	65,80	70	0,62	1,00(0,00-0,00)
Height	1,60(0,07)	1,61(0,07)	36,46	32	0,26	2,66(0,01-6,75)
Family history of congenital anomalies on the mother's side	1,71(0,46)	1,80(0,40)	1,27	1	0,26	1,63(0,69-3,85)
Parents with congenital anomalies*	2,00(0,00)	2,00(0,00)	-	-	-	-
Siblings or grandparents with congenital anomalies	1,88(0,33)	1,98(0,16)	5,44	1	0,02	5,33(4,04-6,56)
Uncles and cousins with congenital anomalies	1,94(0,24)	1,89(0,32)	0,88	1	0,35	0,78(0,11-2,25)
Congenital anomalies in several relatives* Other family members with congenital anomalies Previous pregnancy with anomalies	2,00(0,00)	2,00(0,00)	-	-	-	-
Congenital anomalies in several relatives* Other family members with congenital anomalies Previous pregnancy with anomalies	1,94(0,24)	1,90(0,30)	0,49	1	0,48	0,58(0,12-2,72)
Congenital anomalies in several relatives* Other family members with congenital anomalies Previous pregnancy with anomalies	2,00(0,00)	1,97(0,18)	1,14	1	0,29	2,24×10^-7^(0,00-∞)
Previous miscarriage	1,79(0,41)	1,72(0,45)	0,67	1	0,41	0,68(0,27-1,70)
Adequate prenatal care (considered adequate with more than 6 consultations)	1,09(0,29)	1,03(0,18)	1,91	1	0,17	0,35(0,07-1,65)
Gestational age in weeks at birth	36,30(3,47)	37,42(2,11)	49,52	47	0,37	1,17(1,02-1,35)
Number of pregnancies	2,29(1,66)	2,46(1,25)	20,94	6	< 0,01	1,10(0,82-1,47)
Type of delivery	1,15(0,36)	27,25(288,60)	1,33	3	0,72	1,67(0,61-4,61)
Birth weight	2.596,81(907,17)	3.033,86(638,07)	148,16	142	0,35	1,00(1,00-1,00)
Apgar score at 1° minutes	7,43(2,37)	8,39(0,86)	29,26	10	< 0,01	1,54(1,15-2,05)
Apgar score at 5° minutes	8,57(2,25)	9,53(0,52)	27,95	8	< 0,01	2,19(1,24-3,85)
Fetal status (alive or deceased)	1,15(0,36)	1,02(0,13)	10,70	1	< 0,01	3,16(1,56 a 6,40)
Sex of the child	1,35(0,49)	1,43(0,50)	0,67	1	0,41	8,20(4,95 a 9,16)

SD - standard deviation; X^2^ - chi-square; DF - degrees of freedom; p - significance level; OR - odds ratio; CI - confidence interval. Significance level (p) ≤ 0.05 in bold. There were no cases of parents with congenital anomalies nor anomalies in multiple relatives

Subsequently, Bayesian logistic regression was performed, testing models composed of variables that were found to be significant (p ≤ 0.05) in the chi-square test (X^2^), namely: COVID-19 vaccination during pregnancy, siblings or grandparents with congenital anomalies, number of pregnancies, Apgar score at the 1st minute, Apgar score at the 5th minute, and fetal status. A total of 56 models were compared, with three models showing stronger evidence of the effect between the independent and dependent variables (BF10), accounting for 20% of the variance observed in the data (R^2^): a) Model 1: Siblings or grandparents with congenital anomalies + Apgar score at the 5th minute + fetal status ; b) Model 2: Siblings or grandparents with congenital anomalies + Apgar score at the 1st minute + Apgar score at the 5th minute ; e c) Model 3: Siblings or grandparents with congenital anomalies + number of pregnancies + Apgar score at the 5th minute. The results of Model 1 indicated that the data could predict congenital anomalies 45.8 times (BF10 = 45.80) (Table 3). Next, binary logistic regression (by classification) was performed for the three analyzed models, considering 30% of the variables as test data. When comparing the three models, Model 1 demonstrated the best performance, with the ability to predict 89% of congenital anomaly cases during pregnancy, achieving 90% true positives, 36% false positives, 94% true negatives, and 36% false negatives.

**Table 3:** Bayesian logistic regression and binary logistic regression (by Classification)

Methods	Índices	Model 1	Model 2	Model 3
Bayesian Logistic Regression	BF_10_	45,80	44,09	44,09
	R²	0,20	0,20	0,20
Binary Logistic Regression (by classification)	Accuracy	0,89	0,84	0,80
	Precision (true positive value)	0,90	0,83	0,67
	False positive rate	0,36	0,35	0,51
	True negative value	0,94	0,80	0,41
	False negative rate	0,36	0,35	0,51

Model 1: Siblings or grandparents with congenital anomalies + Apgar score at the 5th minute + fetal status (alive or deceased); Model 2: Siblings or grandparents with congenital anomalies + Apgar score at the 1st minute + Apgar score at the 5th minute; Model 3: Siblings or grandparents with congenital anomalies + number of pregnancies + Apgar score at the 5th minute. BF10, Bayes factor; R², data variance

Subsequently, using the conditional logistic model, the possibility of moderation by the variable «siblings or grandparents with congenital anomalies» was evaluated in relation to other variables in the models (Apgar score at the 1st minute, Apgar score at the 5th minute, fetal status, and number of pregnancies). It was observed that the variable siblings or grandparents with congenital anomalies did not exhibit a moderating effect (p > 0.05) in relation to the other variables analyzed in the models. Furthermore, Model 1 presented the lowest AIC (295.98) and BIC (326.22) values, suggesting a better fit compared to Models 2 and 3 (Table 4).

**Table 4:** Indirect effects (Mediation)

Models		Estimate	DP	p-value	AIC	BIC
Model 1					295,98	326.22
	Apgar at 5th minute → Siblings or grandparents with congenital anomalies → Congenital anomalies	-0.01	0,01	0,80		
	Fetal status → Siblings or grandparents with congenital anomalies → Congenital anomalies	0,01	0,05	0,88		
Model 2					920,44	950,67
	Apgar at 1 minute → Siblings or grandparents with congenital anomalies → congenital anomalies	-0.01	0,01	0,67		
	Apgar at 5 minutes → Siblings or grandparents with congenital anomalies → congenital anomalies	0,01	0,01	0,87		
Model 3					1.079,29	1.109,53
	Apgar at 5 minutes → Siblings or grandparents with congenital anomalies → congenital anomalies	0,01	0,01	0,46		
	Number of pregnancies → Siblings or grandparents with congenital anomalies → congenital anomalies	-0,01	0,01	0,74		

Model 1: Siblings or grandparents with congenital anomalies + Apgar score at the 5th minute + fetal status (alive or deceased); Model 2: Siblings or grandparents with congenital anomalies + Apgar score at the 1st minute + Apgar score at the 5th minute; Model 3: Siblings or grandparents with congenital anomalies + number of pregnancies + Apgar score at the 5th minute. SD, standard deviation; p, significance level; AIC, Akaike Information Criterion; BIC, Bayesian Information Criterion. The SD and p values are based on the delta method

## Discussion

A family history of congenital anomalies, the Apgar score at the 5th minute, and fetal status were identified as factors associated with the presence of congenital anomalies, corroborating findings from previous studies.^(2,4,6)^ Prior research indicates that the presence of congenital anomalies in close family members constitutes a significant risk factor for their occurrence in newborns.^(1,2,5)^ These results reinforce the hypothesis that both genetic factors and conditions related to neonatal vitality may play an important role in increasing the risk of congenital anomalies.

The presence of congenital anomalies in siblings or grandparents is related to the genetic transmission of hereditary conditions that can be passed down from generation to generation.^(2,4,6,18)^ These conditions may involve genetic mutations or specific chromosomal alterations that increase the likelihood of a descendant presenting congenital anomalies.^(19)^ The literature describes that this transmission across generations can occur through Mendelian inheritance, in which the genes responsible for the anomalies may be dominant or recessive, or through multifactorial inheritance, where genetic and environmental factors interact to increase the risk of anomalies.^(20)^ Additionally, consanguinity and marriage between relatives can raise the probability of transmitting recessive conditions, further reinforcing the idea that family history is a significant risk factor.^(2,4,5,21)^

There are few studies in the literature that evaluate the number of pregnancies as a risk factor for the occurrence of congenital anomalies (CAs). However, our data indicate a possible relationship between the number of pregnancies and the occurrence of these anomalies, differing, for example, from a study conducted in China, which showed a reduction in the risk of congenital anomalies as the number of pregnancies increased.^(22)^ On the other hand, the association between the number of pregnancies and congenital anomalies has been reported in the literature, linked to maternal factors (such as age, uterine health, and pre-existing conditions), as well as genetic and environmental factors that may be involved.^(11,18,23–26)^ For example, multiparity (≥3 pregnancies) has been associated with an increased risk of congenital anomalies, possibly due to maternal aging and the accumulation of exposures to environmental and genetic factors over successive pregnancies. There is a need to further explore this relationship between parity and congenital anomalies to gain a better understanding and to identify higher-risk subgroups.^(18,27)^

Other studies have also highlighted similar associations to those found in this study regarding low Apgar scores.^(6,28)^ Consequence of malformation syndromes, it is expected that these babies will exhibit a higher frequency of prematurity due to low birth weight and lower Apgar scores at the 1st and 5th minutes, which contributes to infant morbidity and mortality.^(26)^

In the bivariate analysis, an association was identified between COVID-19 vaccination during pregnancy and the presence of congenital anomalies; however, this relationship should be interpreted with caution.^(29)^ The current literature does not provide robust evidence to support a direct causal relationship between maternal vaccination and the occurrence of anomalies.^(30,31)^ It is possible that confounding factors, such as the characteristics of the studied population or the timing of vaccination, may have influenced the results.

Among the analyzed cases of congenital anomalies, a considerable percentage presented isolated anomalies, meaning a single malformation without other associated alterations, typically caused by multifactorial or sporadic factors. This result was expected, as reported in the literature.^(3,19,32)^ The predominance was in cardiovascular system anomalies, similar to what has been reported in other studies.^(24)^ Among the analyzed cases of congenital anomalies, a considerable percentage presented isolated anomalies, meaning a single malformation without other associated alterations, typically caused by multifactorial or sporadic factors. This result was expected, as described in the literature.^(3,19,32)^ The predominance of cardiovascular system anomalies was consistent with findings from other studies already reported.^(7,24,33)^

This study presents some limitations. The research was conducted in a tertiary-level hospital accredited by the Unified Health System (UHS) for public healthcare services. This circumstance may have influenced the selection of cases and controls, resulting in a sample that does not fully reflect the general population, as women who opted for private prenatal care may have chosen to give birth in private institutions.

Additionally, despite the humanization measures implemented, mothers of the cases may have more detailed recollections of prior exposures (such as medication use, infections, and environmental factors) compared to mothers in the control group, potentially introducing recall bias in data collection. Similarly, self-reported information regarding the number of previous pregnancies and family history may not be entirely accurate.

Another limitation pertains to the study design. As an observational and retrospective case-control study, it can identify associations but cannot establish direct causal relationships. Therefore, although higher parity is linked to an increased risk of congenital anomalies, this does not necessarily imply causation.

Lastly, since the research was conducted at the time of birth, with diagnoses made prior to or during delivery, there may have been underreporting of milder anomalies that were not diagnosed at birth and, therefore, were not included in the analysis.

## Conclusion

The findings reinforce the importance of screening for familial factors and neonatal monitoring as strategies for the early detection and prevention of congenital anomalies. Future studies are needed to explore the observed associations and confirm these results in larger populations.
